# Effects of transcutaneous electrical stimulation on peripheral nerve regeneration after digital neurorrhaphy: A randomized clinical trial.

**DOI:** 10.12688/f1000research.169801.3

**Published:** 2026-05-09

**Authors:** Enilton de Santana Ribeiro de Mattos, Alex Guedes, Yossi Zana, Paulo Itamar Ferraz Lessa, César Romero Antunes Júnior, Eduardo Silva Reis Barreto, Abrahão Fontes Baptista

**Affiliations:** 1 Orthopedics and Traumatology Research Group, Federal University of Bahia (UFBA), Salvador, Bahia, 40110-100, Brazil; 2 Faculty of Medicine of Bahia, Federal University of Bahia, Salvador, Bahia, 40026-010, Brazil; 3 Graduate Program of Neuroscience, Federal University of ABC, São Bernardo do Campo, São Paulo, 09606-045, Brazil; 4 Bahia State University, Salvador, State of Bahia, 41150-000, Brazil; 5 Laboratory of Functional Neuromodulation and Pain, Federal University of Rio de Janeiro (UFRJ), Rio de Janeiro, Rio de Janeiro, 21941-853, Brazil

**Keywords:** Peripheral nerve regeneration, Digital nerve injury, Transcutaneous electrical stimulation, TENS, Randomized controlled trial, Digital nerve

## Abstract

**Background:**

Sensory recovery following digital nerve neurorrhaphy is often incomplete, and strategies to enhance regeneration remain under investigation. Low-frequency transcutaneous electrical stimulation has been proposed as a potential adjunctive therapy, but its efficacy in clinical settings is uncertain.

**Methods:**

In this randomized controlled trial, 32 patients with isolated traumatic digital nerve injuries underwent surgical neurorrhaphy at a tertiary care hospital. Participants were randomly allocated to an intervention group (n = 16) or sham group (n = 16). The intervention consisted of a single postoperative session of square-pulsed, biphasic transcutaneous electrical stimulation at 20 Hz for 1 hour. The sham group received identical conditions without active stimulation. After stimulation, patients underwent physiotherapy sessions for three months. Sensory recovery was assessed using Semmes-Weinstein monofilament testing and two-point discrimination at baseline, 1 week, 1 month, and 3 months postoperatively.

**Results:**

Both groups showed progressive sensory improvement throughout follow-up, approaching normal values at 3 months. No statistically significant differences were observed between groups in any outcome measure. Confidence intervals for group comparisons overlapped, and no clinically meaningful differences were detected. No adverse effects were reported.

**Conclusions:**

In this model, a single postoperative session of low-frequency transcutaneous electrical stimulation did not significantly enhance sensory recovery after digital nerve repair. Further research with varied stimulation protocols, repeated sessions, or extended follow-up may be warranted to clarify its potential role in peripheral nerve regeneration.

**Level of Evidence:**

Therapeutic Level I.

**List of abbreviations**
ANOVA: Analysis of Variance
CSS: Cold Sensitivity Severity Scale
MSW: Modified Semmes-Weinstein
MUNE: Motor Unit Number Estimation
PDI: Pain Disability Index
PES: Peripheral Electrical Stimulation
RCT: Randomized Controlled Trial
s2PD: Static Two-Point Discrimination
SWM: Semmes-Weinstein Monofilament

## Introduction

The human hand is a rich sensory and motor multifunctional tool with dexterous control to perform essential manipulation tasks. ^1^ Peripheral nerve injuries, especially of the upper limb, can result in severe disability and reduced quality of life. ^2– 4^ Several strategies ^5– 8^ including the use of neurotrophic factors, stem cell therapy, ^9, 10^ and electrical stimulation, ^11^ have been investigated to promote peripheral nerve regeneration as well as functional recovery after these traumas. ^12^ Electrical stimulation has also been considered as an ancillary to surgical repair, and its effects on nerve recovery has been the focus of several studies. ^13– 25^

It is to be noted that the characteristics and regenerative potential of peripheral nerves differ markedly depending on the location and type of lesion. ^26^ Differences in digital nerve lesions compared with more proximal and mixed lesions are described. ^26, 27^ Digital nerves are almost exclusively sensory, and injuries to these nerves, properly repaired, generally have shorter regeneration distances and can serve as a model for evaluating the effects of transcutaneous peripheral electrical stimulation (PES), delivered through surface electrodes (commonly referred to as TENS). ^27^ By contrast, proximal nerve injuries, or nerve injuries with larger gaps to overcome, may be more difficult to completely regenerate, given the increased length for axonal growth and the complexity of motor and sensory functional recovery. ^28^

There are different ways to deliver the PES such as implanted electrodes, ^15, 16^ percutaneous electrostimulation ^17, 18^ (acupuncture needles inserted into the skin and connected to an electric current generator), intraoperative electrostimulation, ^19– 23^ thin-film wireless implantable nerve stimulators, ^24^ and surface electrodes. ^25^ The use of transcutaneous surface electrodes is a non-invasive, practical, and simple option, avoiding the reactions provoked by implant surgery or percutaneous stimulation. ^29^

Based on these considerations, we hypothesized that a brief, early postoperative session of transcutaneous PES could modulate biological mechanisms associated with nerve regeneration following digital neurorrhaphy. This hypothesis was grounded on evidence from experimental and clinical studies showing that short-duration electrical stimulation applied soon after nerve repair may enhance activity-dependent regenerative processes. ^23, 30^ In the present study, we intentionally adapted these principles to a non-invasive and clinically feasible protocol, consisting of a single 1-hour session of surface stimulation delivered at motor threshold within 24 hours after surgery. The stimulation protocol (20 Hz, 1 hour) was selected based on the findings of Al-Majed et al. (2000), ^19^ which identified this window as optimal for the upregulation of BDNF and TrkB mRNA. Although the digital nerve is sensory, the intensity was calibrated to the motor threshold of the median nerve to ensuring supramaximal recruitment of large-diameter A-beta sensory fibers, which share similar electrical thresholds with A-alpha motor fibers. Although this approach aimed to reproduce, in a transcutaneous manner, stimulation parameters previously associated with regenerative benefits, its biological effectiveness in purely sensory digital nerves remains unclear. Therefore, we conducted a randomized clinical trial to investigate whether this specific surface PES protocol could influence sensory recovery, cold sensitivity, and pain-related disability in patients undergoing digital nerve repair.

## Methods

This double-blind, randomized, controlled clinical trial was conducted at a general hospital in Bahia, Brazil, from December 19, 2020, to June 10, 2022. The study was prospectively registered in the Brazilian Clinical Trials Registry (ReBEC) on December 18, 2020 (registration number: U1111-1259-1998; available at: https://ensaiosclinicos.gov.br/rg/RBR-8xn3qq5). Ethical approval was obtained from the Research Ethics Committee of the Faculty of Medicine of Bahia, and the study protocol was published ^31^ in advance to ensure methodological transparency and compliance with the Declaration of Helsinki. ^32^

### Participants

Adult patients aged 18 to 60 years with an acute, non-segmental digital nerve injury of the hand were eligible for inclusion if surgical repair was successfully performed within two weeks of injury. Exclusion criteria comprised the presence of metal implants at the surgical site, history of seizures, use of a cardiac pacemaker, local infection or skin lesions at the intervention site, associated bone or tendon injuries, and any pre-existing neuropathies.

### Interventions

All patients underwent standardized microsurgical neurorrhaphy under ultrasound-guided axillary block, with epineural approximation using 2 to 4 nylon 8-0 sutures to align nerve fascicles and minimize trauma. Within 24 hours after surgery, participants were randomly allocated to one of two groups.

The stimulation parameters were chosen based on previous studies related to nerve regeneration and patient safety. ^11, 14, 15, 19^ Group A (Surgery + PES) received one hour of transcutaneous electrical stimulation using the Neurodyn II device (Ibramed, Brazil), delivering a square-pulsed, biphasic, symmetrical current at 20 Hz with a 0.4 ms pulse width. Stimulation intensity was standardized using the median nerve motor threshold as an operational reference, rather than as the anatomical stimulation target. Electrode placement was individualized for the injured digit and the surgical repair site. In all participants, two 1 × 1 cm silicone-carbon gel electrodes were positioned proximal and distal to the neurorrhaphy, along the presumed course of the repaired digital nerve. Therefore, the intervention was anatomically directed to the injured digital nerve branch, although selective and equivalent recruitment of the repaired nerve across all digits cannot be fully confirmed. Group B (Surgery + sham PES) underwent an identical setup with the same device, electrode positioning, and duration, but after an initial perceptible activation, the device output was reduced to zero for the remainder of the session ( Figure 2). A certified physiotherapist, blinded to the group allocation, supervised the rehabilitation protocol. Sessions were remotely monitored via electronic platforms such as WhatsApp or Skype. Patients underwent a hand sensory re-education program based on the approach proposed by Dellon & Jabaley (1982), ^33^ focused on hand sensory re-education over 3-month period. Participants were also encouraged to perform complementary exercises in a home-based program.

**Figure 1.**
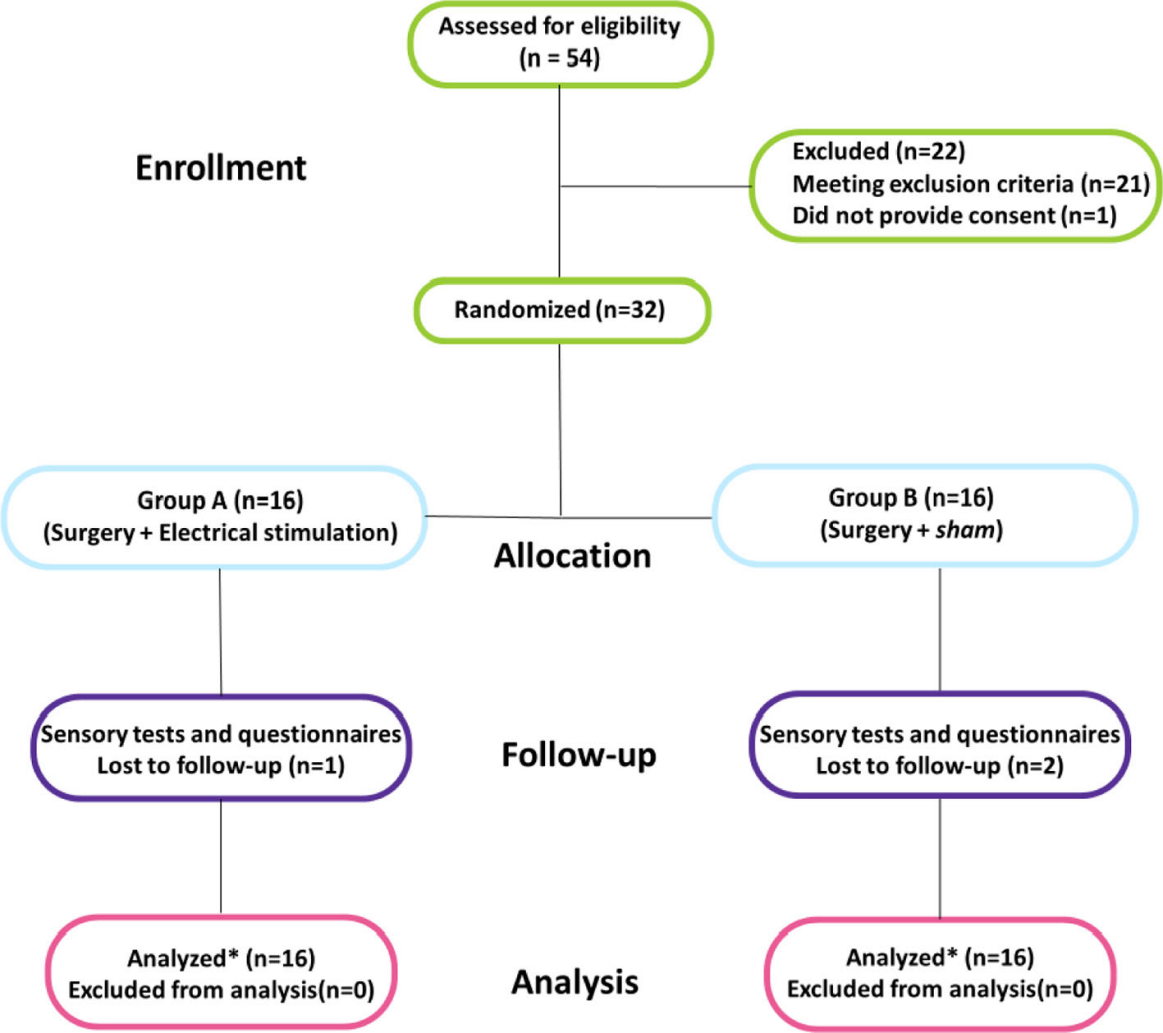
CONSORT 2010 flowchart diagram of patient screening, intervention, and follow-up. *Intention-to-treat (ITT) analysis.

**Figure 2.**
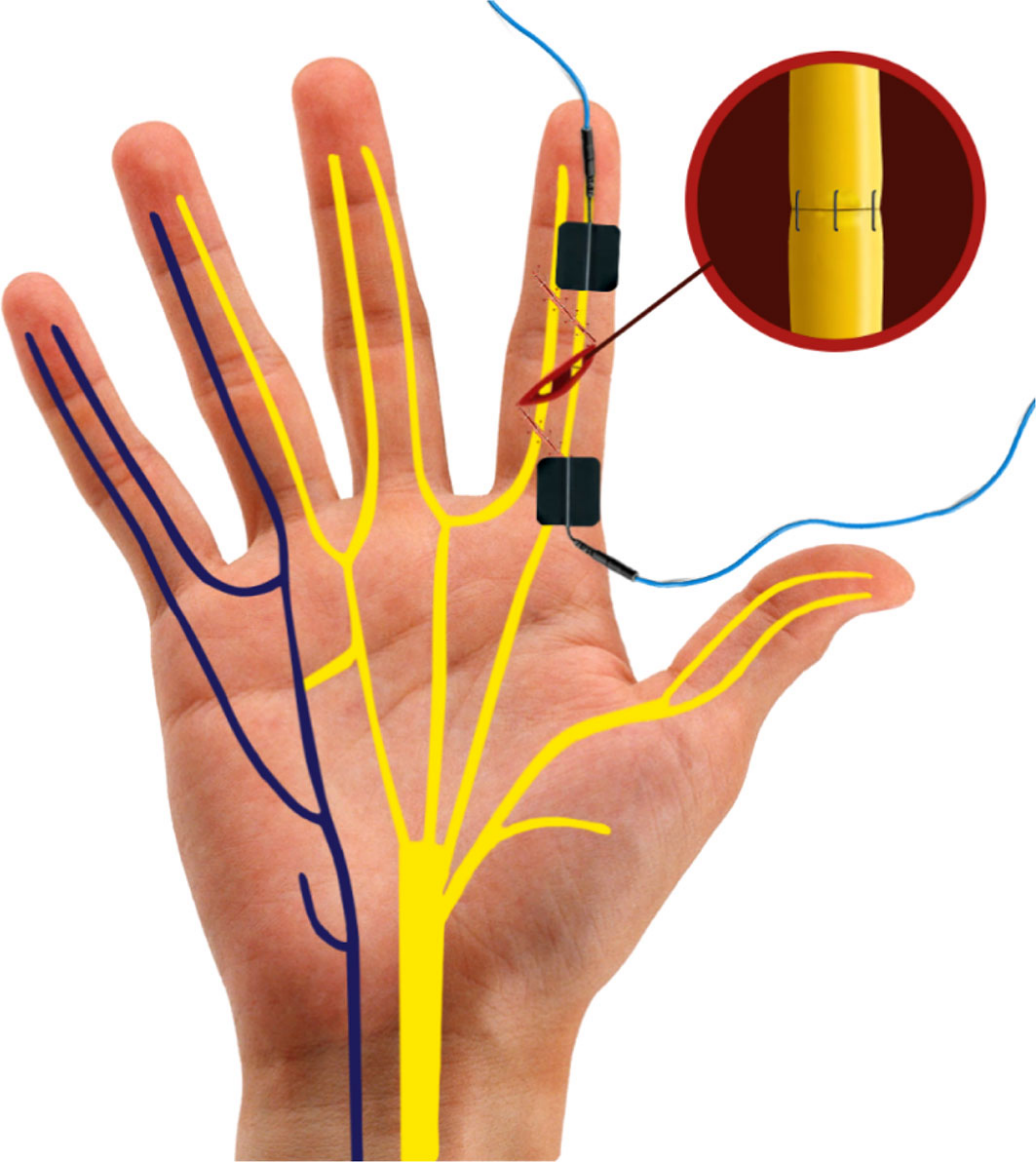
Model of a repaired digital nerve injury of the index finger and the electrodes placement.

### Randomization, allocation concealment, and blinding

Patients were randomly assigned in a 1:1 ratio to Group A (surgery + PES) or Group B (surgery + sham) using an electronic randomization sequence generated with the website randomization.com (available at the time of study planning). Allocation concealment was ensured through centralized management by an independent researcher who was the only person with access to the randomization list. A physiotherapist, blinded to group assignment, administered all stimulation sessions using identical devices with the same electrode placement and duration. For sham sessions, the device was initially activated to produce perceptible stimulation cues before being set to zero output. Although real PES could induce subtle muscle contractions, the identical device design and protocol helped maintain blinding for both participants and the administering physiotherapist.

### Assessment schedule

All patients were evaluated in person by the same surgeon responsible for both the surgical procedure and postoperative follow-up. Assessments were scheduled at four time points: (1) pre-intervention; (2) one-week post-intervention; (3) one-month post-intervention (including ongoing rehabilitation sessions); (4) three months post-intervention (upon completion of all 20 rehabilitation sessions). The three-month follow-up period was selected based on the expected timeframe for peripheral nerve regeneration over short distances (2 to 6 cm), assuming an average axonal growth rate of 1 to 3 mm per day. ^34^

### Outcome measures

The primary outcome was sensory recovery of digital nerves following microsurgical neurorrhaphy, assessed using quantitative sensory tests. Specifically, the Semmes-Weinstein Monofilament (SWM) test and the static two-point discrimination (s2PD) test were applied during four scheduled in-person evaluations. Outcome differences between the two groups (intervention vs. sham) were analyzed post-randomization.

The SWM test, a crucial marker of functional recovery, assesses perception of pressure thresholds related to peripheral reinnervation. ^35^ During the test, participants rested their hands on a table and closed their eyes. In three trials, we applied scored probes perpendicularly to the pulp side of the affected finger for 1 to 1.5 seconds. A positive response in at least two of three trials indicated the sensory threshold. ^23^

The secondary outcome included self-reported measures of cold sensitivity and pain-related functional disability. These were evaluated using the Cold Sensitivity Severity Scale (CSS) ^36^ and the Pain Disability Index (PDI), ^37^ both validated tools for assessing postoperative sensory complaints and pain impact on daily life, aimed to measure improvements in terms of cold sensitivity and pain disability in social functions for individuals who underwent neurorrhaphy of digital nerves in the hand. We used two patient-reported outcome questionnaires: the Cold Sensitivity Severity Scale (CSS) ^36^ and the Pain Disability Index (PDI). ^37^ These patient-reported outcomes were assessed only at the 3-month follow-up, after completion of the rehabilitation protocol.

The s2PD test serves as an established assessment tool for evaluating tactile gnosis. ^2, 38^ It measures the ability to distinguish between two nearby points touching the skin, ensuring they are truly distinct rather than perceived as a single point. The test estimates the minimum distance necessary for the patient to perceive the two pressure points as separate contacts. ^39^ It reflects the degree of innervation in a specific skin area. The Medical Research Council classification, modified by Mackinnon & Dellon, allows grouping based on different value ranges related to the sensitive recovery threshold ^35, 40^ ( Table 2).

**Table 1.** Baseline patient characteristics.

	Electrical stimulation (n = 16)	Sham (n = 16)
Mean age (range in *years)*	36.6 (18–57)	34.2 (21–58)
Male sex ( *no. [%])*	9 (56)	12 (75)
Right-handed ( *no. [%])*	13 (81)	14 (87)
Injury to the dominant hand (no. [%])	6 (38)	8 (50)
Injury to the radial digital nerve ( *no. [%])*	9 (56)	7 (44)
Injured finger		
*Thumb (n=8)*	4	4
*Index (n=9)*	5	4
*Midlle (n=2)*	1	1
*Ringer (n=4)*	1	3
*Litlle (n=9)*	5	4
Diabetes	1	0
Smoker	2	4

**Table 2.** Modified HIGHET classification reproduced from a study by Dunlop *et al.* (2019).

Sensory recovery	Highet	s2PD	m2PD	Recovery of sensibility
Failure	S0			No recovery of sensibility in the autonomous zone of the nerve
Poor	S1			Recovery of deep cutaneous pain sensibility
	S1+			Recovery of superficial pain and some touch sensibility
	S2			Recovery of superficial pain sensibility
	S2+			As with S2, but with over response
	S3	>15 mm	>7 mm	Recovery of pain and touch sensibility with no over response
Good	S3+	7–15 mm	4–7 mm	As in S3, but good localization of the stimulus but imperfect recovery of 2PD
Excellent	S4	2–6 mm	2–3 mm	Complete sensory recovery

The CSS offers a reliable way to assess cold sensitivity. In cases like amputation or nerve damage, hypersensitivity can occur and lead to significant disability. The CSS consists of four questions related to cold-induced symptoms. The total score provides the cold-sensitivity severity score.

The PDI comprises a seven-item questionnaire evaluating how pain affects various aspects of daily life. Each item is rated from 0 (no disability) to 10 (total disability), and the final score (ranging from 0 to 70) reflects the level of disability due to pain. The PDI has demonstrated consistency, validity, and reliability in studies related to nerve damage. ^37^

### Sample size

The sample size was estimated based on effect size data reported by Gordon et al. (2010). ^23^ We calculated the sample size considering a repeated measures analysis of variance (ANOVA) test, accounting for interactions between and within factors. The effect size, as reported by Gordon et al. (2010), ^23^ was 0.26. Additionally, we set an alpha-type error of 5%, a statistical power of 80%, and worked with two groups and three measures.

Adjustments were applied to account for correlations among repeated measures (correlation coefficient = 0.5) and a non-sphericity correction factor of 1.0 (assuming compound symmetry). Based on these assumptions, the minimum required sample was calculated to be 26 participants.

To account for potential losses, we increased the sample size by 20%, resulting in a final sample of 32 patients (16 per group).

### Statistical analysis

The data were evaluated in a paired and non-paired way through within and between-group comparisons. For within-group evaluations, repeated measures ANOVA or the Friedman test was applied, followed by the Student–Newman–Keuls post hoc test. For between-group comparisons, one-way ANOVA or the Kruskal-Wallis test was used, followed by the Student-Newman-Keuls post hoc test. Specifically, repeated measures ANOVA was used for normally distributed SWM and s2PD data across time points, while the Friedman test was applied when normality assumptions were not met. Between-group comparisons at each time point were performed using one-way ANOVA or Kruskal–Wallis tests accordingly. Independent samples t-tests were used for CSS and PDI comparisons at 3 months.

The choice of statistical tests was based on the distribution characteristics of the data, and normality was assessed using the Shapiro-Wilk test or the nature of the data. A 95% confidence interval was considered for statistical analysis, with statistical significance set ai p < 0.05 an alpha of 5% (P < 0.05) and a power of 80%.

Descriptive analysis was conducted using means and standard errors or medians and interquartile range (25 ^th^/75 ^th^ percentiles), as appropriate to data distribution. Both measurements of the variables and the statistical analysis were performed under blinded conditions by assessors unaware of group allocation. The independent variable for both groups was the use of electric current. The dependent variables were derived from the pre- and post-treatment assessments (SWM, s2PD, CSS, and PDI). All statistical tests were performed using JASP (V0.18.3).

## Results

Eligibility was evaluated in a total of 54 patients. Of these, 21 did not meet the inclusion criteria, and one declined to participate. Thus, 32 patients were randomized, with 16 allocated to the PES group and 16 to the sham group ( Figure 1). Baseline characteristics were comparable between groups, and all participants presented with severe sensory impairment on preoperative evaluation, assessed by MSW and s2PD tests ( Table 1). No significant differences between groups were observed during the immediate postoperative period (up to one week; p > 0.05), so subsequent statistical analyses focused on the 1- and 3-month follow-up data.

**Figure 3.**
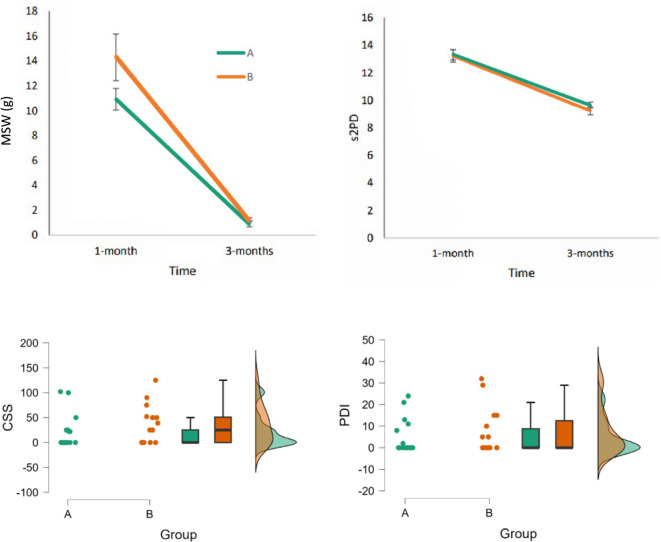
Sensory and patient-reported outcomes after digital nerve repair in the PES and sham groups. Top panels show mean Semmes-Weinstein monofilament thresholds (SWM, grams) and static two-point discrimination (s2PD, millimeters) at 1 and 3 months postoperatively. Both groups demonstrated progressive sensory recovery over time, with no significant differences between PES and sham. Bottom panels show Cold Sensitivity Severity Scale (CSS) and Pain Disability Index (PDI, 0–70) scores at 3 months. Error bars represent standard error of the mean.

Sensory outcomes improved over time in both groups, with significant changes observed between 1 and 3 months postoperatively. Repeated measures ANOVA demonstrated a significant effect of time for both SWM (p = 0.012) and s2PD (p = 0.002), indicating progressive sensory recovery in both groups. No significant group effect or group–time interaction was observed for either outcome (p > 0.05). Age showed a marginal influence on SWM outcomes (p = 0.082), becoming significant when scores were converted to needle size (p = 0.014) ( Figure 3).

As CSS and PDI were assessed only at the 3-month follow-up, comparisons between groups were performed using an independent samples t-test at this single time point. No statistically significant differences were found for CSS (p = 0.305) or PDI (p = 0.477). However, the two scales were highly correlated (r = 0.819, p < 0.001), suggesting consistent subjective perception of disability and cold sensitivity among patients. Age was initially included as a covariate due to its known influence on nerve regeneration. To assess the robustness of the results, sensitivity analyses were performed by (1) excluding age as a covariate and (2) removing an outlier, a 57-year-old participant from the PES group with discrepant SWM values (z-score ≈ 5).

In summary, patients in both groups gradually recovered sensitivity as measured by SWM and s2PD, reaching satisfactory levels at the final assessment. However, no significant treatment effect was found across time points. At 3 months, the unadjusted mean between-group difference between PES and sham was 0.10 g for SWM (95% CI, -0.94 to 1.15) and 0.79 mm for s2PD (95% CI, -0.26 to 1.83). For patient-reported outcomes, the mean between-group difference was -13.71 points for CSS (95% CI, -40.55 to 13.13) and -2.46 points for PDI (95% CI, -9.46 to 4.54) ( Figure 3). Importantly, these sensitivity analyses did not alter the primary conclusions of the study.

## Discussion

This randomized controlled trial investigated the effectiveness of surface PES in promoting sensory recovery following digital nerve neurorrhaphy. Both groups exhibited a gradual recovery of sensory function over the 3-month follow-up, as measured by SWM and s2PD tests. From a theoretical perspective, electrical stimulation is thought to enhance nerve regeneration through activity-dependent mechanisms that require effective recruitment of regenerating axons and sufficient current density at the nerve site. In the present study, the transcutaneous delivery of stimulation, applied at motor threshold in a purely sensory nerve injury, may not have provided the selectivity or intensity of fiber activation necessary to trigger these mechanisms. ^41^ Although age showed a near-significant effect in some models, its overall influence appears limited. Cold sensitivity and pain-related disability were assessed only at the final follow-up and showed high inter-individual variability, limiting the ability to draw definitive conclusions regarding these secondary outcomes. Transcutaneous electrical stimulation holds promise in nerve regeneration, offering a non-invasive approach with potential practical benefits. ^42^ It can be utilized to circumvent the complications of surgical implantation or percutaneous stimulation. ^43, 44^

Some research indicates that it may take up to 8 weeks for the regenerating axons to cover a distance of 25mm, and the use of PES may reduce this period. ^20, 26^ Previous results demonstrated that subjects who received stimulation exhibited earlier and better outcomes around 3 months post-surgery. ^26^ Gordon at al. (2010) ^23^ conducted an innovative randomized controlled trial (RCT) of 21 patients undergoing carpal tunnel decompression surgery. Postoperative direct nerve stimulation using implanted wires (20 Hz, 4-6 volts 0.1-0.8 ms) for one hour led to earlier improvements in electrophysiological parameters when compared to controls. Simillarly, Wong et al. (2015) ^26^ conducted a double-blind RCT involving 31 patients with transected digital nerves and observed significantly improved sensory outcomes with PES (20 Hz, <30 V, 0.1–0.4 ms), although no differences in overall functional recovery were found. In another trial, Power et al. (2020) ^45^ evaluated PES following cubital tunnel decompression in 31 patients. The intervention group received a single 1-hour session of PES (20 Hz, <30 V, 0.1 ms) and demonstrated greater improvements in Motor Unit Number Estimation (MUNE) over time compared to the control group. Importantly, these studies employed direct, implanted, or intraoperative stimulation, ensuring higher current density and selective activation of nerve fibers at the repair site, conditions that differ substantially from transcutaneous stimulation and may explain the discrepancy in findings. The present findings should also be interpreted in light of the absence of a dose–response framework. This trial tested a single 1-hour session of surface PES, selected to mirror parameters previously associated with regenerative effects in experimental and clinical studies. However, neuromodulatory effects may depend on stimulation dose, timing, repetition, current density, and cumulative exposure. Therefore, the absence of benefit with this single-session protocol does not exclude the possibility that repeated sessions, different intensities, alternative timing, or longer stimulation schedules could produce different outcomes.

Some studies reported adverse findings that contradict prior research that has highlighted the advantageous impact of direct current electric fields on the regeneration of peripheral nerves. ^46, 47^ In the present trial, neither the s2PD nor the SWM tests demonstrated a significant enhancement in tactile receptor reinnervation in the digital pulp among patients who received PES. Given these results, the effectiveness of transcutaneous applied electrical fields in promoting in vivo peripheral nerve regeneration remains uncertain.

Cold intolerance ^48^ and pain ^49^ are commonly reported sources of substantial morbidity following nerve injuries in the hand. Previous studies have associated the severity of these symptoms with the degree of sensory recovery, with poorer reinnervation correlating with more pronounced functional impairment and discomfort. ^49, 50^ In the present sample, isolated digital nerve injuries were not associated with worse outcomes in terms of pain or cold intolerance, as measured by the CSS and PDI. These symptoms appear to be more commonly linked to complex cases involving finger replantation, severe vascular compromise, or proximal nerve injuries of the median or ulnar nerves.

Postoperative rehabilitation following hand neurorrhaphy is considered standard of care ^51^ and was prescribed for all participants in this study. Withholding rehabilitation would be ethically inappropriate, as hand therapy is routinely recommended after surgery in real-world clinical settings. Omission of such care could also compromise the study’s external validity. Nevertheless, the use of structured rehabilitation in both groups may have reduced the ability to isolate the specific contribution of PES. Because sensory re-education and hand therapy are expected to promote functional recovery after neurorrhaphy, improvements driven by rehabilitation in both groups may have masked subtle additive effects of the stimulation protocol. ^52^

The use of a digital nerve injury model in this study presents inherent limitations regarding the generalizability of the findings. Digital nerve injuries differ substantially from proximal or mixed nerve lesions, particularly due to the shorter axonal regeneration distances and the absence of motor components. Because isolated digital nerve injuries typically show rapid and favorable recovery after repair, the short regeneration distance may have produced a ceiling effect by three months. In addition, although SWM and s2PD are widely used and clinically meaningful tools after digital nerve repair, they may have limited sensitivity to detect subtle differences in axonal regeneration, receptor reinnervation, or sensory processing. ^53– 55^ Therefore, small biological effects of PES may have remained below the detection threshold of these clinical measures. Consequently, although our findings provide insight into the effects of surface PES in isolated digital sensory nerve injuries, they may not be directly applicable to more clinically challenging scenarios, such as mixed motor-sensory nerve injuries, delayed repairs, segmental defects, nerve gaps, or proximal lesions requiring longer regenerative distances. Future studies should consider the use of more complex clinical models, such as proximal or mixed nerve injuries, to better assess the potential of electrical stimulation in promoting meaningful functional recovery. Therefore, the present findings should be interpreted as evidence regarding the limitations of this specific stimulation paradigm in this clinical model, rather than as evidence against the potential usefulness of electrical stimulation as a broader therapeutic strategy for peripheral nerve regeneration.

### Study limitations

One limitation of PES is the potential interference of the anesthetic technique with its effectiveness. Ideally, surgery should be performed under general anesthesia. A recent study in rats ^56^ demonstrated that the perioperative use of lidocaine significantly reduced the beneficial effects of electrical stimulation on nerve regeneration. In our study, an axillary block (far from the stimulation site) was used, and PES was applied after post-anesthetic recovery. However, a potential attenuating effect on nerve due to anesthesia cannot be completely ruled out. Current trends in hand surgery increasingly favor local or regional anesthesia, given their advantages of lower perioperative risk, faster recovery, and superior postoperative analgesia. Therefore, proposing PES as an adjunct treatment in digital nerve repair under general anesthesia (without first evaluating its use under standard anesthetic conditions) may limit its clinical applicability.

Skin impedance may have resulted in insufficient current density reaching the nerve when compared to the levels achieved with implanted electrodes. ^23^ In addition, the short regeneration distance in isolated digital nerve injuries may have contributed to a ceiling effect, while SWM and s2PD may not have been sensitive enough to detect subtle treatment-related differences.

The 3-month follow-up also limits the temporal resolution of the findings. This period captures early sensory recovery after short-distance digital nerve repair, but later outcomes at 6 to 12 months may better reflect sensory refinement, cortical reorganization, functional integration, and patient adaptation during daily hand use.

Another limitation of this study is that part of the functional evaluation relied on subjective, patient-reported data. Although neurophysiological assessments can serve as sensitive tools for evaluating the severity and progression of nerve injuries in adults, their application was limited in this study. All patients had isolated digital nerve lacerations, which complicates electrophysiological interpretation due to signal contamination through volume conduction from the intact digital nerve branch on the opposite side of the finger. ^26^ Nevertheless, recent outcome research in the field of peripheral nerve injury has increasingly emphasized the importance of combining functional assessments with patient-reported outcomes. ^57^

## Conclusion

The specific protocol of surface PES tested in this study may not confer additional clinical benefit in isolated digital nerve injuries under standard rehabilitation conditions. No significant differences were observed in cold sensitivity or pain-related disability outcomes between the intervention and control groups. These findings suggest that, within the context of isolated digital nerve injuries, surface PES may not confer additional clinical benefit beyond standard surgical repair and rehabilitation. Further research is warranted to explore the efficacy and safety of electrical stimulation protocols in more complex nerve injury models and under varying clinical conditions.

## Ethical considerations

This study was approved by the Research Ethics Committee of the Faculty of Medicine of Bahia (Federal University of Bahia), under protocol number 4.430.230. All procedures were conducted in accordance with the Declaration of Helsinki. The study was prospectively registered in the Brazilian Clinical Trials Registry (ReBEC) under registration number U1111-1259-1998.

## Consent to participate

All participants provided written informed consent prior to enrollment in the study, including consent for surgical intervention, application of surface PES, and participation in follow-up assessments.

## Data Availability

All data underlying the results of this study are available within the article itself. The CONSORT 2010 checklist and flowchart associated with this trial are openly available in Figshare at https://doi.org/10.6084/m9.figshare.30120691, under the Creative Commons CC0 license. ^58^ The database and project analysis are also openly available in Figshare under the Creative Commons CC0 license at https://doi.org/10.6084/m9.figshare.30350653. ^59^ Additional study resources are available in Figshare, including the list of study materials https://doi.org/10.6084/m9.figshare.13636685.v1, ^60^ the available facilities https://doi.org/10.6084/m9.figshare.13636694.v1, ^61^ and the participant information sheet and consent form. Reporting guidelines of protocol are also available in Figshare, specifically the SPIRIT checklist for “Influence of surface peripheral electrical stimulation on nerve regeneration after digital nerve neurorrhaphy: study protocol for a randomized clinical trial”. ^62^ All data and materials are available under the terms of the Creative Commons Zero “No rights reserved” data waiver (CC0 1.0 Public domain dedication).

## References

[1] Liu M-J Xiong C-H Xiong L : Biomechanical Characteristics of Hand Coordination in Grasping Activities of Daily Living. *PLoS One.* 2016;11:e0146193. 10.1371/journal.pone.0146193 26730579 PMC4701170

[2] Dunlop RLE Wormald JCR Jain A : Outcome of surgical repair of adult digital nerve injury: a systematic review. *BMJ Open.* 2019;9:e025443. 10.1136/bmjopen-2018-025443 30872549 PMC6429897

[3] Stoll G Müller HW : Nerve Injury, Axonal Degeneration and Neural Regeneration: Basic Insights. *Brain Pathol.* 1999;9:313–325. 10.1111/j.1750-3639.1999.tb00229.x 10219748 PMC8098499

[4] Rosén B Lundborg G : A model instrument for the documentation of outcome after nerve repair. *J. Hand Surg. Am.* 2000;25:535–543. 10.1053/jhsu.2000.6458 10811759

[5] Crisci AR Ferreira AL : Low-intensity pulsed ultrasound accelerates the regeneration of the sciatic nerve after neurotomy in rats. *Ultrasound Med. Biol.* 2002;28:1335–1341. 10.1016/S0301-5629(02)00576-8 12467860

[6] Bae C-S Lim S-C Kim K-Y : Effect of Ga-as laser on the regeneration of injured sciatic nerves in the rat. *In Vivo.* 2004;18:489–495. 15369190

[7] Molteni R Zheng J-Q Ying Z : Voluntary exercise increases axonal regeneration from sensory neurons. *Proc. Natl. Acad. Sci.* 2004;101:8473–8478. 10.1073/pnas.0401443101 15159540 PMC420418

[8] Seo TB Han I Yoon J-H : Involvement of Cdc2 in Axonal Regeneration Enhanced by Exercise Training in Rats. *Med. Sci. Sports Exerc.* 2006;38:1267–1276. 10.1249/01.mss.0000227311.00976.68 Reference Source 16826023

[9] Pereira Lopes FR Camargo de Moura Campos L Dias Corrêa J : Bone marrow stromal cells and resorbable collagen guidance tubes enhance sciatic nerve regeneration in mice. *Exp. Neurol.* 2006;198:457–468. 10.1016/j.expneurol.2005.12.019 16487971

[10] Tohill M Terenghi G : Stem-cell plasticity and therapy for injuries of the peripheral nervous system. *Biotechnol. Appl. Biochem.* 2004;40:17–24. 10.1042/BA20030173 15270703

[11] Geremia NM Gordon T Brushart TM : Electrical stimulation promotes sensory neuron regeneration and growth-associated gene expression. *Exp. Neurol.* 2007;205:347–359. 10.1016/j.expneurol.2007.01.040 17428474

[12] Güvenç K Ateş E Mert Asfuroğlu Z : Factors affecting functional outcomes after surgery to repair extensive volar forearm lacerations with nerve injuries identified via quantitative and qualitative methods. *Jt Dis. Relat. Surg.* 2023;34:405–412. 10.52312/jdrs.2023.1067 37462645 PMC10367148

[13] Baptista AF Gomes JR d S Oliveira JT : A new approach to assess function after sciatic nerve lesion in the mouse—Adaptation of the sciatic static index. *J. Neurosci. Methods.* 2007;161:259–264. 10.1016/j.jneumeth.2006.11.016 17204334

[14] Baptista AF Gomes JRS Oliveira JT : High- and low-frequency transcutaneous electrical nerve stimulation delay sciatic nerve regeneration after crush lesion in the mouse. *J. Peripher. Nerv. Syst.* 2008;13:71–80. 10.1111/j.1529-8027.2008.00160.x 18346233

[15] Mendonça AC Barbieri CH Mazzer N : Directly applied low intensity direct electric current enhances peripheral nerve regeneration in rats. *J. Neurosci. Methods.* 2003;129:183–190. 10.1016/S0165-0270(03)00207-3 14511820

[16] Politis MJ Zanakis MF Albala BJ : Facilitated Regeneration in the Rat Peripheral Nervous System Using Applied Electric Fields. *The Journal of Trauma: Injury, Infection, and Critical Care.* 1988;28:1375–1381. 10.1097/00005373-198809000-00012 Reference Source 3262170

[17] Rodriguez Lagos L Arribas-Romano A Fernández-Carnero J : Effects of Percutaneous and Transcutaneous Electrical Nerve Stimulation on Endogenous Pain Mechanisms in Patients with Musculoskeletal Pain: A Systematic Review and Meta-Analysis. *Pain Med.* 2023;24:397–414. 10.1093/pm/pnac140 36130064

[18] Inoue M Hojo T Yano T : The Effects of Electroacupuncture on Peripheral Nerve Regeneration in Rats. *Acupunct. Med.* 2003;21:9–17. 10.1136/aim.21.1-2.9 12924841

[19] Al-Majed AA Brushart TM Gordon T : Electrical stimulation accelerates and increases expression of BDNF and trkB mRNA in regenerating rat femoral motoneurons. *Eur. J. Neurosci.* 2000;12:4381–4390. 10.1046/j.1460-9568.2000.01341.x 11122348

[20] Al-Majed AA Neumann CM Brushart TM : Brief Electrical Stimulation Promotes the Speed and Accuracy of Motor Axonal Regeneration. *J. Neurosci.* 2000;20:2602–2608. 10.1523/JNEUROSCI.20-07-02602.2000 10729340 PMC6772244

[21] Al-Majed AA Tam SL Gordon T : Electrical Stimulation Accelerates and Enhances Expression of Regeneration-Associated Genes in Regenerating Rat Femoral Motoneurons. *Cell. Mol. Neurobiol.* 2004;24:379–402. 10.1023/B:CEMN.0000022770.66463.f7 15206821 PMC11529956

[22] Brushart TM Hoffman PN Royall RM : Electrical Stimulation Promotes Motoneuron Regeneration without Increasing Its Speed or Conditioning the Neuron. *J. Neurosci.* 2002;22:6631–6638. 10.1523/JNEUROSCI.22-15-06631.2002 12151542 PMC6758126

[23] Gordon T Amirjani N Edwards DC : Brief post-surgical electrical stimulation accelerates axon regeneration and muscle reinnervation without affecting the functional measures in carpal tunnel syndrome patients. *Exp. Neurol.* 2010;223:192–202. 10.1016/j.expneurol.2009.09.020 19800329

[24] Ray WZ Mahan MA Guo D : An update on addressing important peripheral nerve problems: challenges and potential solutions. *Acta Neurochir.* 2017;159:1765–1773. 10.1007/s00701-017-3203-3 28500566

[25] Chandran P Sluka KA : Development of opioid tolerance with repeated transcutaneous electrical nerve stimulation administration. *Pain.* 2003;102:195–201. 10.1016/s0304-3959(02)00381-0 12620611

[26] Wong JN Olson JL Morhart MJ : Electrical stimulation enhances sensory recovery: A randomized controlled trial. *Ann. Neurol.* 2015;77:996–1006. 10.1002/ana.24397 25727139

[27] Hardy PB Wang BY Chan KM : The use of electrical stimulation to enhance recovery following peripheral nerve injury. *Muscle Nerve.* 2024;70:1151–1162. 10.1002/mus.28262 39347555

[28] Costello MC Errante EL Smartz T : Clinical applications of electrical stimulation for peripheral nerve injury: a systematic review. *Front. Neurosci.* 2023;17. 10.3389/fnins.2023.1162851 PMC10435250 37600003

[29] Russell C Roche AD Chakrabarty S : Peripheral nerve bionic interface: a review of electrodes. *Int. J. Intell. Robot. Appl.* 2019;3:11–18. 10.1007/s41315-019-00086-3

[30] Park S Liu CY Ward PJ : Effects of Repeated 20-Hz Electrical Stimulation on Functional Recovery Following Peripheral Nerve Injury. *Neurorehabil. Neural Repair.* 2019;33(9):775–784. 10.1177/1545968319862563 31328654 PMC6693960

[31] Mattos E Guedes A Lessa PIF : Influence of surface peripheral electrical stimulation on nerve regeneration after digital nerve neurorrhaphy: study protocol for a randomized clinical trial. *F1000Res.* 2021;10:219. 10.12688/f1000research.42120.1 34909180 PMC8596177

[32] World Medical Association Declaration of Helsinki. *JAMA.* 2013;310:2191. 10.1001/jama.2013.281053 24141714

[33] Dellon AL Jabaley ME : Reeducation of sensation in the hand following nerve suture. *Clin. Orthop. Relat. Res.* 1982;163:75–79. 10.1097/00003086-198203000-00011 7067267

[34] Sunderland S : RATE OF REGENERATION IN HUMAN PERIPHERAL NERVES. *Arch. Neurol. Psychiatr.* 1947;58:251–295. 10.1001/archneurpsyc.1947.02300320002001 20265595

[35] Wang Y Sunitha M Chung KC : How to Measure Outcomes of Peripheral Nerve Surgery. *Hand Clin.* 2013;29:349–361. 10.1016/j.hcl.2013.04.004 23895715 PMC3746316

[36] McCabe SJ Mizgala C Glickman L : The measurement of cold sensitivity of the hand. *J. Hand Surg. Am.* 1991;16:1037–1040. 10.1016/S0363-5023(10)80065-6 1748748

[37] Novak CB Anastakis DJ Beaton DE : Relationships Among Pain Disability, Pain Intensity, Illness Intrusiveness, and Upper Extremity Disability in Patients With Traumatic Peripheral Nerve Injury. *J. Hand Surg. Am.* 2010;35:1633–1639. 10.1016/j.jhsa.2010.07.018 20888499

[38] Shooter D : Use of two-point discrimination as a nerve repair assessment tool: preliminary report. *ANZ J. Surg.* 2005;75(10):866–868. 10.1111/j.1445-2197.2005.03557.x 16176227

[39] Craig J Johnson K : The two-point threshold. *Curr. Dir. Psychol. Sci.* 2000;9:29–32. 10.1111/1467-8721.00054

[40] Slutsky DJ : The Management of Digital Nerve Injuries. *J. Hand Surg. Am.* 2014;39:1208–1215. 10.1016/j.jhsa.2013.12.012 24862117

[41] Liu Y Fox PM : The Role of Electrical Stimulation in Peripheral Nerve Regeneration: Current Evidence and Future Directions. *J. Hand Surg. Glob. Online.* 2024;6(5):718–721. Published 2024 Mar 27. 10.1016/j.jhsg.2024.01.022 39381378 PMC11456620

[42] Juckett L Saffari TM Ormseth B : The Effect of Electrical Stimulation on Nerve Regeneration Following Peripheral Nerve Injury. *Biomolecules.* 2022;12:1856. 10.3390/biom12121856 36551285 PMC9775635

[43] Burssens P Forsyth R Steyaert A : Influence of burst TENS stimulation on collagen formation after Achilles tendon suture in man. A histological evaluation with Movat’s pentachrome stain. *Acta Orthop. Belg.* 2005;71:342–346. 16035709

[44] Burssens P Forsyth R Steyaert A : Influence of burst TENS stimulation on the healing of Achilles tendon suture in man. *Acta Orthop. Belg.* 2003;69:528–532. 14748110

[45] Power HA Morhart MJ Olson JL : Postsurgical Electrical Stimulation Enhances Recovery Following Surgery for Severe Cubital Tunnel Syndrome: A Double-Blind Randomized Controlled Trial. *Neurosurgery.* 2020;86:769–777. 10.1093/neuros/nyz322 31432080

[46] McGinnis ME Murphy DJ : The lack of an effect of applied d.c. electric fields on peripheral nerve regeneration in the guinea pig. *Neuroscience.* 1992;51:231–244. 10.1016/0306-4522(92)90488-N 1465183

[47] Hansom SM McGinnis ME : Regeneration of rat sciatic nerves in silicone tubes: Characterization of the response to low intensity d.c. stimulation. *Neuroscience.* 1994;58:411–421. 10.1016/0306-4522(94)90047-7 8152547

[48] Irwin MS Gilbert SEA Terenghi G : Cold Intolerance Following Peripheral Nerve Injury. *J. Hand Surg.* 1997;22:308–316. 10.1016/S0266-7681(97)80392-0 9222907

[49] Miclescu A Straatmann A Gkatziani P : Chronic neuropathic pain after traumatic peripheral nerve injuries in the upper extremity: prevalence, demographic and surgical determinants, impact on health and on pain medication. *Scand. J. Pain.* 2019;20:95–108. 10.1515/sjpain-2019-0111 31536038

[50] Aman M Zimmermann KS Glaser JJ : Revealing digital nerve lesions–A comprehensive analysis of 2084 cases of a specialized center. *Injury.* 2024;55:111514. 10.1016/j.injury.2024.111514 38555200

[51] Chan T Ho C Lee W : Functional Outcome of the Hand following Flexor Tendon Repair at the ‘No Man’s Land’. *J. Orthop. Surg.* 2006;14:178–83. 10.1177/230949900601400214 16914784

[52] Royston P Sauerbrei W : Two Techniques for Investigating Interactions between Treatment and Continuous Covariates in Clinical Trials. *The Stata Journal: Promoting communications on statistics and Stata.* 2009;9:230–251. 10.1177/1536867X0900900204

[53] Willand MP Nguyen M-A Borschel GH : Electrical Stimulation to Promote Peripheral Nerve Regeneration. *Neurorehabil. Neural Repair.* 2016;30:490–496. 10.1177/1545968315604399 26359343

[54] Carey-Ewend A Goldfarb J Randall Z : The current state of outcome measurements after peripheral nerve injury: a systematic review. *J. Hand Surg. Glob. Online.* 2025;7:192–195. 10.1016/j.jhsg.2024.12.001 40182860 PMC11962904

[55] Keane GC Marsh EB Hunter DA : Lidocaine Nerve Block Diminishes the Effects of Therapeutic Electrical Stimulation to Enhance Nerve Regeneration in Rats. *Hand.* 2023;18:119S–125S. 10.1177/15589447221093668 35579211 PMC9896284

[56] Heneghan C Goldacre B Mahtani KR : Why clinical trial outcomes fail to translate into benefits for patients. *Trials.* 2017;18:122. 10.1186/s13063-017-1870-2 28288676 PMC5348914

[57] Saarinen A Pakarinen O Vaajala M : Randomized controlled trials reporting patient-reported outcomes with no significant differences between study groups are potentially susceptible to unjustified conclusions—a systematic review. *J. Clin. Epidemiol.* 2024;169:111308. 10.1016/j.jclinepi.2024.111308 38428542

[58] Mattos E d SR de Guedes A Zana Y : CONSORT 2010 Checklist for “Effects of Transcutaneous Electrical Stimulation on Peripheral Nerve Regeneration after Digital Neurorrhaphy: A Randomized Clinical Trial”. 2025 [cited 2025 Sep 28]. 10.6084/m9.figshare.30120691 PMC13250519 42282261

[59] Mattos E d SR de Guedes A Lessa PIF : Database and analysis of the RCT. 2025 [cited 2025 Out 13]. 10.6084/m9.figshare.30350653

[60] Mattos E d SR de Guedes A Lessa PIF : List of materials study number U1111-1259-1998. 2021 [cited 2025 Sep 28]. 10.6084/m9.figshare.13636685

[61] Mattos ESR de Guedes A Lessa PIF : Available facilities study number U1111-1259-1998. 2021 [cited 2025 Sep 28]. 10.6084/m9.figshare.13636694

[62] Mattos E d SR de Guedes A Lessa PIF : SPIRIT-Checklist-for randomised studies protocols. 2021 [cited 2025 Sep 28]. 10.6084/m9.figshare.13584764.v1

