# Supplementary material for: Effects of transcutaneous electrical stimulation on peripheral nerve regeneration after digital neurorrhaphy: A randomized clinical trial
Source: F1000Res. 2026 Sep 22;14:1222. Originally published 2025 Nov 6. [Version 4] doi: 10.12688/f1000research.169801.4 (PMC13250519; doi:10.12688/f1000research.169801.4)
Supplement: Peer review history

# Reviewer report: Reviewer response for version 4

**DOI:** 10.5256/f1000research.204088.r523744

**Published:** 2026-09-24

**Authors:** Johannes Heinzel

**Affiliations:** 1 Department of Hand-, Plastic, Reconstructive and Burn Surgery, BG Klinik Tuebingen, University of Tuebingen, Tuebingen, Baden-Wuerttemberg, Germany

No additional comments.

Is the work clearly and accurately presented and does it cite the current literature?

Yes

If applicable, is the statistical analysis and its interpretation appropriate?

Yes

Are all the source data underlying the results available to ensure full reproducibility?

Yes

Is the study design appropriate and is the work technically sound?

Yes

Are the conclusions drawn adequately supported by the results?

Yes

Are sufficient details of methods and analysis provided to allow replication by others?

Yes

Reviewer Expertise:

nerve regeneration, nerve surgery, functional recovery

I confirm that I have read this submission and believe that I have an appropriate level of expertise to confirm that it is of an acceptable scientific standard.

# Reviewer report: Reviewer response for version 3

**DOI:** 10.5256/f1000research.200497.r486394

**Published:** 2026-06-09

**Authors:** Christoph A Schroen

**Affiliations:** 1 Icahn School of Medicine at Mount Sinai, New York, New York, USA; 2 Universitat Heidelberg Medizinische Fakultat Heidelberg, Heidelberg, Baden-Württemberg, Germany

Thank you for the opportunity to review this manuscript. This study presents a well-conducted and highly relevant randomized clinical trial investigating whether a single 1-hour postoperative session of transcutaneous electrical nerve stimulation following digital nerve neurorrhaphy may improve sensory recovery compared to a sham group. Both groups improved significantly at 3 months, although no differences between groups were observed in any outcome measure. The authors conclude that this protocol of transcutaneous nerve stimulation may thus not enhance sensory recovery after digital nerve repair. 

 I believe this study addresses a timely and important research question, and the methodology used in this study is appropriate for answering this question. Nonetheless, I believe a few suggestions and considerations should be addressed:

 - The placement of both electrodes may warrant additional discussion. The nerve injuries in this study are very distal, and Wallerian degeneration may extend further proximally than the position of the proximal electrode. If true, this would mean that a more proximal electrode may have been more efficacious at improving nerve recovery. The overall great outcomes at 3 months in both groups suggest that Wallerian degeneration remained limited to an area close to the repair site, but additional considerations regarding electrode placement may improve the discussion of this study. 

 - In the results section, the authors mention that no signification between group differences were observed, but they do not present the specific p-values. Instead, they state "p > 0.05". Please add the exact p-values for between-group comparisons at all three time points to the results section. 

 - In this study, both groups demonstrate overall great outcomes at three months postoperatively. In previous studies, electrical stimulation has been shown to accelerate functional recovery and improve outcomes after peripheral nerve injury. The authors are appropriately cautious when interpreting the results from this study and provide a thoughtful discussion including potential reasons for the discordance between their findings and previous literature. Nonetheless, as both groups recovered significantly, these results can be explained by a ceiling effect, which should be addressed in the limitations section as a limitation in patient selection and study design.

 - If possible, please discuss briefly whether electrical stimulation may be more helpful for motor recovery or sensory recovery or similarly beneficial, including potential reasons.

 - In Figure 3, the labels should explicitly state whether data is from the treatment or sham group instead of only stating "A" or "B". Please make it more obvious which dataset belongs to which group.

 Addressing the points above may improve the interpretability of these findings. Overall, this is a well-conducted study that adds meaningfully to the literature.

Is the work clearly and accurately presented and does it cite the current literature?

Yes

If applicable, is the statistical analysis and its interpretation appropriate?

Yes

Are all the source data underlying the results available to ensure full reproducibility?

Yes

Is the study design appropriate and is the work technically sound?

Yes

Are the conclusions drawn adequately supported by the results?

Yes

Are sufficient details of methods and analysis provided to allow replication by others?

Yes

Reviewer Expertise:

Peripheral Nerve Injury; Nerve Injury Animal Models; Basic Science; Electrical Nerve Stimulation; Intraoperative Nerve Imaging; Orthopaedic Surgery Research.

I confirm that I have read this submission and believe that I have an appropriate level of expertise to confirm that it is of an acceptable scientific standard, however I have significant reservations, as outlined above.

# Response

**Published:** 2026-06-16

**Authors:** Eduardo Silva Reis Barreto

**Affiliations:** School of Medicine, Universidade Federal da Bahia, Salvador, State of Bahia, Brazil

Review report: “Effects of transcutaneous electrical stimulation on peripheral nerve regeneration after digital neurorrhaphy: A randomized clinical trial”.

 June 15, 2026

*Dear Editor and Reviewers,*

*Thank you for your valuable feedback, which has greatly improved the quality and clarity of our manuscript. We have made the requested changes and are available for any further revisions. Below are our responses to the reviewers' comments:*

**#Reviewer 3:**

 Thank you for the opportunity to review this manuscript. This study presents a well-conducted and highly relevant randomized clinical trial investigating whether a single 1-hour postoperative session of transcutaneous electrical nerve stimulation following digital nerve neurorrhaphy may improve sensory recovery compared to a sham group. Both groups improved significantly at 3 months, although no differences between groups were observed in any outcome measure. The authors conclude that this protocol of transcutaneous nerve stimulation may thus not enhance sensory recovery after digital nerve repair.

 I believe this study addresses a timely and important research question, and the methodology used in this study is appropriate for answering this question. Nonetheless, I believe a few suggestions and considerations should be addressed:

 (1) The placement of both electrodes may warrant additional discussion. The nerve injuries in this study are very distal, and Wallerian degeneration may extend further proximally than the position of the proximal electrode. If true, this would mean that a more proximal electrode may have been more efficacious at improving nerve recovery. The overall great outcomes at 3 months in both groups suggest that Wallerian degeneration remained limited to an area close to the repair site, but additional considerations regarding electrode placement may improve the discussion of this study.

 (2) In the results section, the authors mention that no signification between group differences were observed, but they do not present the specific p-values. Instead, they state "p > 0.05". Please add the exact p-values for between-group comparisons at all three time points to the results section.

 (3) In this study, both groups demonstrate overall great outcomes at three months postoperatively. In previous studies, electrical stimulation has been shown to accelerate functional recovery and improve outcomes after peripheral nerve injury. The authors are appropriately cautious when interpreting the results from this study and provide a thoughtful discussion including potential reasons for the discordance between their findings and previous literature. Nonetheless, as both groups recovered significantly, these results can be explained by a ceiling effect, which should be addressed in the limitations section as a limitation in patient selection and study design.

 (4) If possible, please discuss briefly whether electrical stimulation may be more helpful for motor recovery or sensory recovery or similarly beneficial, including potential reasons.

 (5) In Figure 3, the labels should explicitly state whether data is from the treatment or sham group instead of only stating "A" or "B". Please make it more obvious which dataset belongs to which group.

 Addressing the points above may improve the interpretability of these findings. Overall, this is a well-conducted study that adds meaningfully to the literature.

**Author response and action taken:** *We thank the reviewer for the careful evaluation of our manuscript and for the constructive comments. Below, we address each point and summarize the changes made in the revised version.*

1. *We agree with the reviewer that electrode placement is an important aspect of the stimulation protocol. We added a paragraph to the Discussion addressing the possibility that the local transcutaneous montage around the neurorrhaphy may not have optimally recruited the relevant proximal axonal segment or produced sufficient current density across the repair zone. We also discuss that electrode position and field geometry should be considered active components of dose delivery rather than merely technical details.*
2. *The Results section was revised to include the exact p-values for between-group comparisons and for the repeated-measures ANOVA group and group-by-time effects.*
3. *We agree that the favorable recovery observed in both groups may have reduced the ability to detect an additive effect of PES. We revised the Study limitations section to explicitly state that selecting isolated distal digital nerve injuries may have introduced a ceiling effect due to the short regeneration distance and the absence of a motor component.*
4. *A new paragraph was added to the Discussion to address differences between motor, mixed, and purely sensory nerve injury models and their implications for detecting the effects of electrical stimulation.*
5. *Figure 3 and its legend were revised to clarify which data correspond to the PES group and the sham group.*

# Reviewer report: Reviewer response for version 3

**DOI:** 10.5256/f1000research.200497.r483123

**Published:** 2026-05-14

**Authors:** Johannes Heinzel

**Affiliations:** 1 Department of Hand-, Plastic, Reconstructive and Burn Surgery, BG Klinik Tuebingen, University of Tuebingen, Tuebingen, Baden-Wuerttemberg, Germany

The authors have sufficiently addressed my concerns. I endorse publication of this manuscript.

Is the work clearly and accurately presented and does it cite the current literature?

Yes

If applicable, is the statistical analysis and its interpretation appropriate?

Yes

Are all the source data underlying the results available to ensure full reproducibility?

Yes

Is the study design appropriate and is the work technically sound?

Yes

Are the conclusions drawn adequately supported by the results?

Yes

Are sufficient details of methods and analysis provided to allow replication by others?

Yes

Reviewer Expertise:

nerve regeneration, nerve surgery, functional recovery

I confirm that I have read this submission and believe that I have an appropriate level of expertise to confirm that it is of an acceptable scientific standard.

# Reviewer report: Reviewer response for version 2

**DOI:** 10.5256/f1000research.196634.r474927

**Published:** 2026-04-21

**Authors:** Johannes Heinzel

**Affiliations:** 1 Department of Hand-, Plastic, Reconstructive and Burn Surgery, BG Klinik Tuebingen, University of Tuebingen, Tuebingen, Baden-Wuerttemberg, Germany

This manuscript presents a well-conducted randomized controlled trial addressing a clinically relevant question in peripheral nerve regeneration. The study is methodologically rigorous, clearly reported, and provides valuable negative findings in a field where such data remain underrepresented. I would like to offer several additional comments that may further strengthen the manuscript:

- **External validity and patient selection:** The inclusion of isolated, non-segmental digital nerve injuries represents a relatively favorable clinical scenario with inherently good recovery potential. While this is acknowledged, it would be helpful to more explicitly discuss how this limits generalizability to more clinically challenging cases (e.g., mixed nerves, delayed repair, or segmental defects), where adjunctive therapies may be more impactful.
- **Dose–response considerations:** The study investigates a single stimulation session. Expanding the discussion to more explicitly address the absence of a dose–response framework would help contextualize the negative findings within the broader neuromodulation literature.
- **Rehabilitation as a potential confounder:** All patients underwent structured postoperative physiotherapy, which is appropriate and ethically required. However, given its known impact on sensory recovery, a more explicit discussion of its potential to mask subtle intervention effects would be valuable.
- **Temporal resolution of outcomes:** With follow-up limited to 3 months, the study primarily captures early sensory recovery. It would be helpful to briefly discuss whether later time points (e.g., 6–12 months), particularly for cortical reorganization and functional integration, might reveal different effects.
- **Clinical relevance of endpoints:** While SWM and s2PD are established measures, a short comment on how observed changes translate into patient-relevant functional outcomes (e.g., dexterity, daily hand use) would further strengthen the clinical interpretation. The use of PROMs could also be discussed. 

Overall, this is a high-quality study with a clear clinical message. Addressing the points above would further enhance its interpretability and clinical applicability.

Is the work clearly and accurately presented and does it cite the current literature?

Yes

If applicable, is the statistical analysis and its interpretation appropriate?

Yes

Are all the source data underlying the results available to ensure full reproducibility?

Yes

Is the study design appropriate and is the work technically sound?

Yes

Are the conclusions drawn adequately supported by the results?

Yes

Are sufficient details of methods and analysis provided to allow replication by others?

Yes

Reviewer Expertise:

nerve regeneration, nerve surgery, functional recovery

I confirm that I have read this submission and believe that I have an appropriate level of expertise to confirm that it is of an acceptable scientific standard, however I have significant reservations, as outlined above.

# Response

**Published:** 2026-04-27

**Authors:** Eduardo Silva Reis Barreto

**Affiliations:** School of Medicine, Universidade Federal da Bahia, Salvador, State of Bahia, Brazil

We thank the reviewer for the positive and constructive assessment of our manuscript. We have revised the Discussion and Study limitations sections to address the main points raised.

 First, we expanded the discussion of external validity to clarify that isolated, non-segmental digital nerve injuries represent a relatively favorable sensory nerve injury model. We now state more explicitly that the findings may not be directly generalizable to more clinically challenging scenarios, such as mixed motor-sensory nerve injuries, delayed repairs, segmental defects, nerve gaps, or proximal lesions requiring longer regenerative distances.

 Second, we added a discussion of dose–response considerations. We now clarify that this trial tested a single 1-hour session of surface PES and therefore cannot exclude the possibility that repeated sessions, different intensities, alternative timing, or longer stimulation schedules could produce different effects.

 Third, we expanded the discussion of postoperative rehabilitation. We emphasize that structured sensory re-education and hand therapy were ethically appropriate and clinically necessary, but may have promoted recovery in both groups and thereby reduced the ability to detect subtle additive effects of PES.

 Fourth, we added a limitation regarding the 3-month follow-up. We now state that this period primarily captures early sensory recovery after short-distance digital nerve repair and that later assessments at 6 to 12 months may better reflect sensory refinement, cortical reorganization, functional integration, and patient adaptation during daily hand use.

 Regarding the clinical relevance of the endpoints, we agree with the reviewer’s point. This issue is now addressed in the revised Discussion together with the related concern raised by Reviewer 1 regarding the sensitivity of SWM and s2PD. We clarify that although SWM and s2PD are established and clinically meaningful tools after digital nerve repair, they may not fully capture subtle treatment-related differences or broader patient-relevant functional outcomes. We also note that CSS and PDI provided patient-reported information on cold sensitivity and pain-related disability, although broader hand-specific PROMs and dexterity-based or daily hand-use assessments may be useful in future studies. Accordingly, the null findings should be interpreted as the absence of a detectable clinical advantage of this protocol, rather than definitive evidence of no biological effect.

# Reviewer report: Reviewer response for version 2

**DOI:** 10.5256/f1000research.196634.r466700

**Published:** 2026-04-03

**Authors:** Nish Mohith Kurukuti

**Affiliations:** 1 Northwestern University, Evanston, Illinois, USA

I appreciate the authors’ thorough and thoughtful revisions. The manuscript has improved substantially in terms of theoretical clarity, methodological transparency, and interpretation of null findings. Overall, the manuscript is improved. However, I have a few targeted suggestions that would further strengthen the paper.

 I remain unclear regarding how the stimulation intervention was anatomically targeted across participants. The manuscript states that PES was delivered “at the motor threshold of the median nerve,” while Table 1 indicates injuries across multiple digits, including the ring and little fingers, which may involve digital branches outside the median nerve distribution. It is therefore not clear whether stimulation was standardized to the injured digital nerve branch, or whether a more general median nerve-based stimulation approach was used across all participants.

 This distinction is important because the biological plausibility of the intervention depends on whether the injured nerve was actually engaged in a comparable manner across subjects.

 While the authors state that *“small effect sizes and overlapping confidence intervals”* are shown in Figure 3, numerical values are still not explicitly reported in the text or tables. Null results are best interpreted via precision (CI width) and effect magnitude, not just p-values. Furthermore, readers should not have to visually infer this from plots. I would recommend adding a concise table or sentence reporting mean difference (PES vs sham), 95% CI, Effect size (e.g., Cohen’s d or partial η² for ANOVA)

 The ceiling effect discussion is now adequate. However, I think going a step further with also addressing the sensitivity of the outcome measures would also be helpful. I suggest discussing the possibility that the current chosen outcome measures may not detect small differences and Implication for interpretation of null results

Is the work clearly and accurately presented and does it cite the current literature?

Partly

If applicable, is the statistical analysis and its interpretation appropriate?

Yes

Are all the source data underlying the results available to ensure full reproducibility?

Yes

Is the study design appropriate and is the work technically sound?

Yes

Are the conclusions drawn adequately supported by the results?

Yes

Are sufficient details of methods and analysis provided to allow replication by others?

Partly

Reviewer Expertise:

TENS, sensorimotor control, Sensory perception, Motor control, Motor unit physiology

I confirm that I have read this submission and believe that I have an appropriate level of expertise to confirm that it is of an acceptable scientific standard, however I have significant reservations, as outlined above.

# Response

**Published:** 2026-04-27

**Authors:** Eduardo Silva Reis Barreto

**Affiliations:** School of Medicine, Universidade Federal da Bahia, Salvador, State of Bahia, Brazil

We thank the reviewer for the thoughtful and constructive comments, and for recognizing the improvements made in the revised manuscript. We agree that the anatomical targeting of the stimulation protocol required further clarification. The Methods section has been revised to specify that electrode placement was individualized according to the injured digit and surgical repair site, with electrodes positioned proximal and distal to the neurorrhaphy along the presumed course of the repaired digital nerve. We also clarified that the median nerve motor threshold was used only as an operational reference to standardize stimulation intensity, not as the anatomical target of stimulation. To avoid overstatement, we now acknowledge that selective and equivalent recruitment of the repaired digital nerve branch across all digits cannot be fully confirmed.

 We also agree that null findings are better interpreted through the magnitude of between-group differences and precision rather than p-values alone. Accordingly, we revised the Results section to include numerical between-group estimates in the original clinical units. Specifically, we added the unadjusted mean differences and 95% confidence intervals for SWM, s2PD, CSS, and PDI at the 3-month assessment. We chose this format rather than standardized effect sizes because mean differences in grams, millimeters, and questionnaire points are more directly interpretable in a clinical trial context. These estimates did not indicate a clear advantage of PES over sham.

 Finally, we revised the Discussion and Study limitations sections to address the sensitivity of the outcome measures. We now clarify that, although SWM and s2PD are clinically established tools after digital nerve repair, they may not detect subtle treatment-related differences in axonal regeneration, receptor reinnervation, or sensory processing. We also emphasize that this limitation, together with the possible ceiling effect by three months, should be considered when interpreting the null findings.

# Reviewer report: Reviewer response for version 1

**DOI:** 10.5256/f1000research.187178.r448494

**Published:** 2026-01-24

**Authors:** Nish Mohith Kurukuti

**Affiliations:** 1 Northwestern University, Evanston, Illinois, USA

This manuscript reports a double-blind randomized controlled trial evaluating whether a single postoperative session of low-frequency transcutaneous peripheral electrical stimulation (PES) improves sensory recovery after digital nerve neurorrhaphy. The topic is clinically relevant and timely, particularly given ongoing interest in non-invasive neuromodulation strategies to enhance peripheral nerve regeneration. The study is carefully conducted, prospectively registered, and transparently reported, and it contributes valuable negative/null findings to a literature that is otherwise skewed toward positive results.

 However, while the methodological execution is generally sound, the manuscript would benefit from clearer justification of the stimulation paradigm, stronger alignment between hypotheses and outcomes, and more critical interpretation of null effects, particularly in light of biological plausibility, ceiling effects, and protocol design choices. Several issues related to statistical reporting, outcome sensitivity, and figure/table presentation should be addressed to improve clarity and scientific impact.

 Overall, I believe the study is suitable for indexing after moderate revisions.

 Major Comments:

 1. Rationale and biological plausibility of the stimulation protocol

 The manuscript would benefit from a more explicit justification for the choice of a single 1-hour PES session, particularly when many preclinical and clinical studies suggesting benefit have used either direct nerve stimulation, repeated sessions, or different timing relative to injury and repair.

 While the authors cite prior work using single-session stimulation, the mechanistic assumptions underlying transcutaneous stimulation at motor threshold—especially in a purely sensory digital nerve—are not sufficiently discussed.

 The Discussion should more clearly distinguish failure of PES as a modality from failure of the specific PES protocol tested.

 2. Sensitivity and ceiling effects of outcome measures

 The use of SWM and s2PD testing is clinically standard; however, in isolated digital nerve injuries with short regeneration distances, these measures may lack sensitivity to detect incremental benefits.

 By 3 months, both groups approach near-normal sensory thresholds, suggesting a ceiling effect. Secondary outcomes (CSS and PDI) were assessed only at the final time point, limiting their interpretability as recovery trajectories.

 The authors should more explicitly frame this as a model-limited sensitivity issue, and discuss whether alternative outcomes (e.g., dynamic 2PD, tactile gnosis tasks, neurophysiology, or longer follow-up) might have been more informative.

 3. Statistical analysis and reporting clarity

 Several aspects of the statistical reporting require clarification:

 The rationale for switching between parametric and non-parametric tests is described, but results are not always clearly tied to specific tests.

 Confidence intervals are mentioned conceptually but are not consistently reported numerically in the Results or figures.

 The handling of age as a covariate, and the post hoc removal of an outlier, are described but could be perceived as analytically fragile without clearer justification.

 Explicitly report effect sizes and confidence intervals for primary outcomes.

 Clear indication (in text or tables) of which statistical tests were used for each comparison.

 A brief clarification that sensitivity analyses did not alter the primary conclusions.

 4. Interpretation of null results and conclusions

 The conclusions are appropriately cautious but could be strengthened by a more theory-driven interpretation.

 The Discussion sometimes implies uncertainty about PES efficacy in general, whereas the data more specifically speak to surface PES in isolated digital nerve repair under standard rehabilitation.

 The comparison to studies using implanted or intraoperative stimulation should emphasize differences in current density, fiber recruitment, and timing.

 Refine the Conclusions to explicitly state that the findings apply to this specific clinical context and stimulation paradigm, rather than PES broadly.

 Minor Comments:

1. Figures and captions: Figure captions should be more standalone and include key take-home messages, particularly for Figure 3. Axis labels and units should be consistently legible and clearly explained without reference to the main text.
2. Terminology consistency: The manuscript alternates between PES, TENS, and surface electrical stimulation. While related, these terms should be clearly defined and used consistently.
3. Secondary outcomes timing: The limitation of assessing CSS and PDI only at 3 months should be stated more prominently earlier in the Results or Methods.

Is the work clearly and accurately presented and does it cite the current literature?

Partly

If applicable, is the statistical analysis and its interpretation appropriate?

Yes

Are all the source data underlying the results available to ensure full reproducibility?

Yes

Is the study design appropriate and is the work technically sound?

Yes

Are the conclusions drawn adequately supported by the results?

Yes

Are sufficient details of methods and analysis provided to allow replication by others?

Partly

Reviewer Expertise:

TENS, sensorimotor control, Sensory perception, Motor control, Motor unit physiology

I confirm that I have read this submission and believe that I have an appropriate level of expertise to confirm that it is of an acceptable scientific standard, however I have significant reservations, as outlined above.

# Response

**Published:** 2026-02-13

**Authors:** Eduardo Silva Reis Barreto

**Affiliations:** School of Medicine, Universidade Federal da Bahia, Salvador, State of Bahia, Brazil

Review report: “Effects of transcutaneous electrical stimulation on peripheral nerve regeneration after digital neurorrhaphy: A randomized clinical trial”.

 February 2026

*Dear Editor and Reviewers,*

*Thank you for your valuable feedback, which has greatly improved the quality and clarity of our manuscript. We have made the requested changes and are available for any further revisions. Below are our responses to the reviewers' comments:*

**#Reviewer 1:**

 This manuscript reports a double-blind randomized controlled trial evaluating whether a single postoperative session of low-frequency transcutaneous peripheral electrical stimulation (PES) improves sensory recovery after digital nerve neurorrhaphy. The topic is clinically relevant and timely, particularly given ongoing interest in non-invasive neuromodulation strategies to enhance peripheral nerve regeneration. The study is carefully conducted, prospectively registered, and transparently reported, and it contributes valuable negative/null findings to a literature that is otherwise skewed toward positive results.

 However, while the methodological execution is generally sound, the manuscript would benefit from clearer justification of the stimulation paradigm, stronger alignment between hypotheses and outcomes, and more critical interpretation of null effects, particularly in light of biological plausibility, ceiling effects, and protocol design choices. Several issues related to statistical reporting, outcome sensitivity, and figure/table presentation should be addressed to improve clarity and scientific impact.

 Overall, I believe the study is suitable for indexing after moderate revisions.

 Major Comments:

 1. Rationale and biological plausibility of the stimulation protocol

 The manuscript would benefit from a more explicit justification for the choice of a single 1-hour PES session, particularly when many preclinical and clinical studies suggesting benefit have used either direct nerve stimulation, repeated sessions, or different timing relative to injury and repair

 While the authors cite prior work using single-session stimulation, the mechanistic assumptions underlying transcutaneous stimulation at motor threshold—especially in a purely sensory digital nerve—are not sufficiently discussed.

 The Discussion should more clearly distinguish failure of PES as a modality from failure of the specific PES protocol tested.

**Author response and action taken: **We thank the reviewer for this valuable and insightful comment. In response, we revised the final paragraph of the Introduction to more clearly explain the biological rationale behind the specific stimulation protocol used in this study. We clarified why a single early postoperative session of transcutaneous stimulation at motor threshold was selected, how this choice was inspired by prior experimental and clinical evidence using brief stimulation paradigms, and why the applicability of this approach to purely sensory digital nerves remains uncertain. These changes also help distinguish the absence of effect observed in this trial from a general conclusion about electrical stimulation as a therapeutic modality, emphasizing that our findings refer specifically to the protocol tested.

 2. Sensitivity and ceiling effects of outcome measures

 The use of SWM and s2PD testing is clinically standard; however, in isolated digital nerve injuries with short regeneration distances, these measures may lack sensitivity to detect incremental benefits.

 By 3 months, both groups approach near-normal sensory thresholds, suggesting a ceiling effect. Secondary outcomes (CSS and PDI) were assessed only at the final time point, limiting their interpretability as recovery trajectories.

 The authors should more explicitly frame this as a model-limited sensitivity issue, and discuss whether alternative outcomes (e.g., dynamic 2PD, tactile gnosis tasks, neurophysiology, or longer follow-up) might have been more informative.

**Author response and action taken: **We thank the reviewer for this thoughtful and constructive comment. In response, we clarified in the Discussion that the use of isolated digital nerve injuries as a clinical model may have limited the sensitivity of the outcome measures, since the short regeneration distance and favorable natural recovery after repair likely produced a ceiling effect by three months, reducing the ability of standard tests such as SWM and s2PD to detect small incremental differences between groups. We also acknowledged that secondary outcomes (CSS and PDI), assessed only at the final time point, do not allow interpretation of recovery trajectories, and we added a brief consideration that alternative assessments and longer follow-up might have provided greater sensitivity to detect subtle effects.

 3. Statistical analysis and reporting clarity

 Several aspects of the statistical reporting require clarification:

 (1) The rationale for switching between parametric and non-parametric tests is described, but results are not always clearly tied to specific tests.

 (2) Confidence intervals are mentioned conceptually but are not consistently reported numerically in the Results or figures.

 (3) The handling of age as a covariate, and the post hoc removal of an outlier, are described but could be perceived as analytically fragile without clearer justification.

 (4) Explicitly report effect sizes and confidence intervals for primary outcomes.

 (5) Clear indication (in text or tables) of which statistical tests were used for each comparison.

 (6) A brief clarification that sensitivity analyses did not alter the primary conclusions.

**Author response and action taken: **We thank the reviewer for these important observations regarding the clarity of the statistical reporting. In response, we revised the Statistical analysis and Results sections to explicitly link each reported result to the corresponding statistical test, clearly indicating where repeated measures ANOVA, non-parametric alternatives, and independent samples tests were applied. We also clarified the rationale for including age as a covariate and reframed the outlier analysis as a planned sensitivity analysis to assess the robustness of the findings, rather than a post hoc adjustment. The Results were edited to explicitly state the absence of group and group–time interaction effects, and to indicate that effect sizes and overlapping confidence intervals for the primary outcomes are presented in Figure 3. Finally, we added a clear statement confirming that the sensitivity analyses did not alter the primary conclusions of the study.

 4. Interpretation of null results and conclusions

 (1) The conclusions are appropriately cautious but could be strengthened by a more theory-driven interpretation.

 (2) The Discussion sometimes implies uncertainty about PES efficacy in general, whereas the data more specifically speak to surface PES in isolated digital nerve repair under standard rehabilitation.

 (3) The comparison to studies using implanted or intraoperative stimulation should emphasize differences in current density, fiber recruitment, and timing.

 (4) Refine the Conclusions to explicitly state that the findings apply to this specific clinical context and stimulation paradigm, rather than PES broadly.

**Author response and action taken: **We thank the reviewer for this thoughtful and insightful comment. In response, we refined the Discussion to provide a more theory-driven interpretation of the null findings, emphasizing the mechanisms by which electrical stimulation is believed to promote nerve regeneration and clarifying how transcutaneous stimulation at motor threshold in a purely sensory digital nerve may not achieve the current density or selective fiber recruitment described in studies using direct or implanted stimulation. We also expanded the comparison with prior clinical trials to explicitly highlight differences in stimulation delivery, timing, and biological plausibility. Finally, we revised the Conclusion to state more precisely that the findings apply to the specific surface PES protocol tested in this clinical context, rather than to electrical stimulation as a therapeutic modality in general.

 Minor Comments:

 Figures and captions: Figure captions should be more standalone and include key take-home messages, particularly for Figure 3. Axis labels and units should be consistently legible and clearly explained without reference to the main text.

**Author response and action taken: **We thank the reviewer for this helpful suggestion. In response, we revised the caption of Figure 3 to make it more self-contained and informative, including explicit descriptions of the variables, units of measurement, and the main findings illustrated in the figure. We also reviewed the axis labels to ensure clarity and consistency, allowing the figure to be interpreted independently from the main text.

 Terminology consistency: The manuscript alternates between PES, TENS, and surface electrical stimulation. While related, these terms should be clearly defined and used consistently.

**Author response and action taken: **We thank the reviewer for this important observation. We revised the manuscript to ensure consistent terminology throughout the text. The term transcutaneous peripheral electrical stimulation (PES) is now clearly defined at first mention as stimulation delivered through surface electrodes (commonly referred to as TENS), and the abbreviation PES is used consistently thereafter to avoid ambiguity.

 Secondary outcomes timing: The limitation of assessing CSS and PDI only at 3 months should be stated more prominently earlier in the Results or Methods.

**Author response and action taken: **We thank the reviewer for this helpful suggestion. In response, we clarified in both the Methods and Results sections that CSS and PDI were assessed only at the 3-month follow-up, making this temporal limitation explicit earlier in the manuscript rather than only in the Discussion.
